# Correction: Engineering surface state density of monolayer CVD grown 2D MoS_2_ for enhanced photodetector performance

**DOI:** 10.1371/journal.pone.0316118

**Published:** 2024-12-17

**Authors:** Gowtham Polumati, Chandra Sekhar Reddy Kolli, Andres de Luna Bugallo, Parikshit Sahatiya

The affiliation for the third and fourth author is incorrect. Andres de Luna Bugallo is not affiliated with #2 but with #3: Centro de Física Aplicada y Tecnología Avanzada, Universidad Nacional Autónoma de México, A.P. 1–1010, Querétaro, Qro., México. Parikshit Sahatiya is not affiliated with #3 but with #2: Materials Center for Sustainable Energy & Environment, Birla Institute of Technology and Science Pilani, Hyderabad Campus, Hyderabad, India.
