# Supplementary figures and images for: Engineering surface state density of monolayer CVD grown 2D MoS2 for enhanced photodetector performance
Source: PLoS One. 2024 Apr 10;19(4):e0297825. doi: 10.1371/journal.pone.0297825 (PMC11006120; doi:10.1371/journal.pone.0297825)

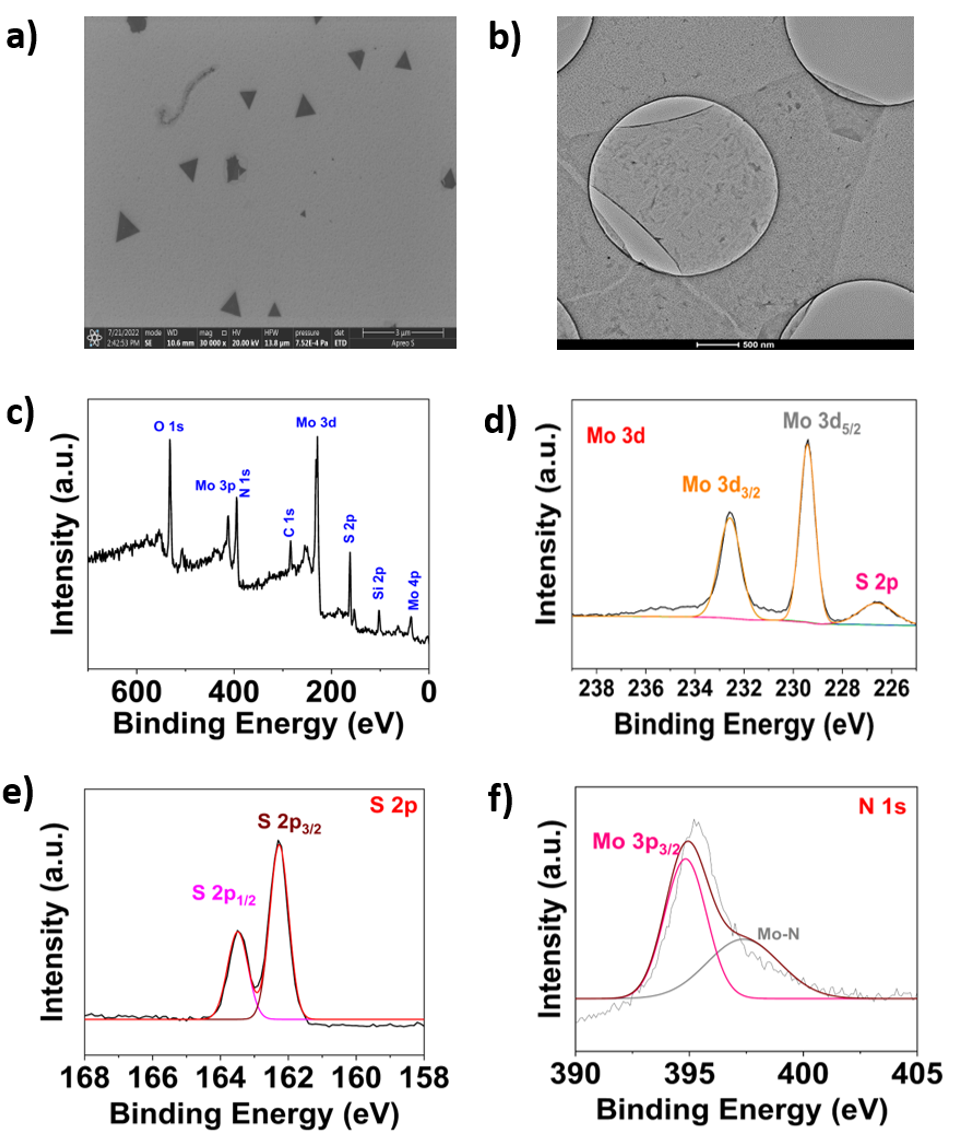

Supplement: S1 Fig — a) FESEM images of nitrogen doped MoS2. b) HRTEM image of monolayer MoS2 c) XPS survey spectra of N-doped MoS2, d), e) & f) Individual high-resolution XPS spectra of Mo 3d, S 2p & N 1s of nitrogen doped MoS2. (TIF) [file pone.0297825.s001.tif]

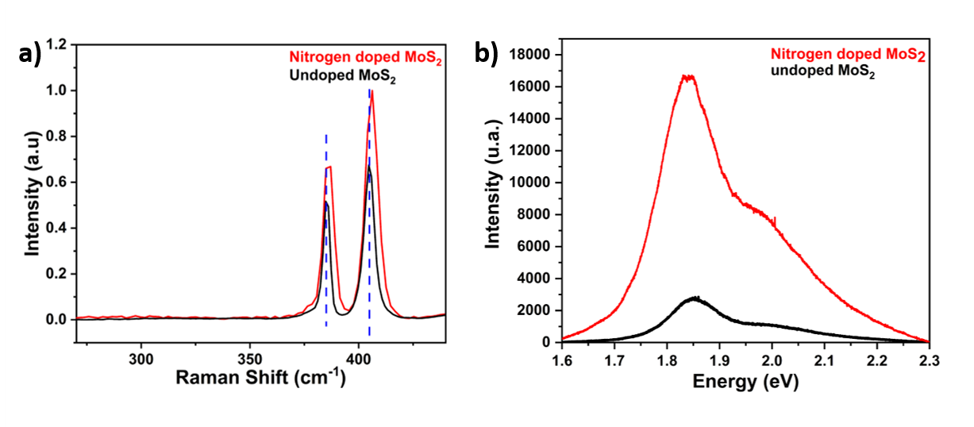

Supplement: S2 Fig — a) The Raman and b) PL spectra of monolayer MoS2 that has been nitrogen-doped and undoped on a SiO2/Si substrate. (TIF) [file pone.0297825.s002.tif]

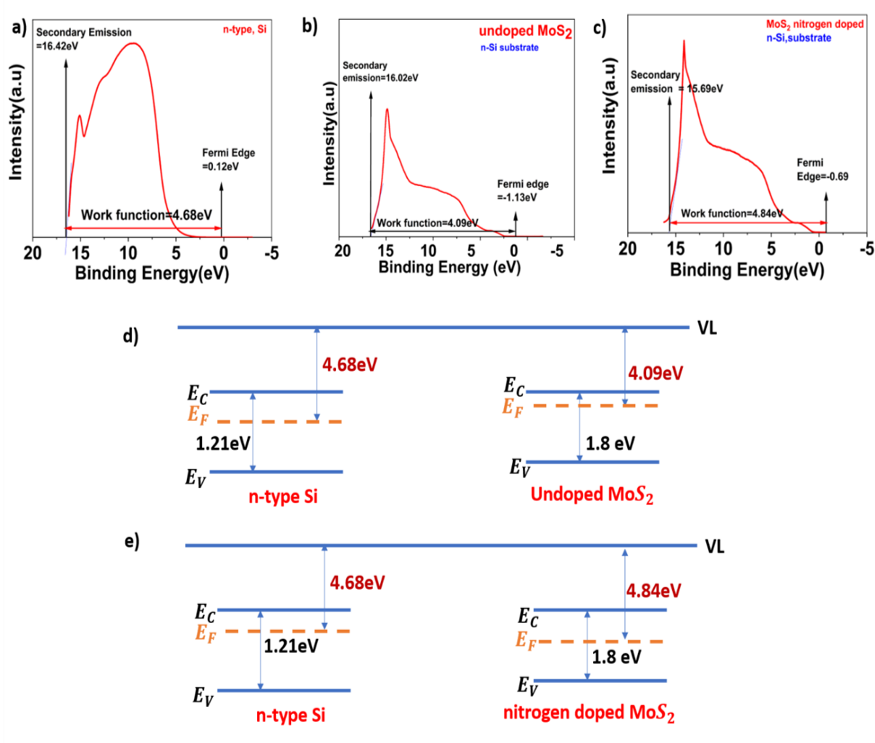

Supplement: S3 Fig — a-b-c) UPS spectra (measured by He I source, hν = 21.22 eV) of n-type silicon substrate, undoped MoS2 and nitrogen doped MoS2 grown on n-type substrate. c)Schematic representation of energy-band diagram n-type of silicon substrate and undoped MoS2 in equilibrium condition (when isolated) d) energy-band diagram n-type of silicon substrate and nitrogen doped MoS2 in equilibrium condition (when isolated). (TIF) [file pone.0297825.s003.tif]
