# Supplementary material for: Engineering surface state density of monolayer CVD grown 2D MoS2 for enhanced photodetector performance
Source: PLoS One. 2024 Apr 10;19(4):e0297825. doi: 10.1371/journal.pone.0297825 (PMC11006120; doi:10.1371/journal.pone.0297825)
Supplement: Attachment — Submitted filename: Plos On comments New.docx [file pone.0297825.s004.docx]

Bugallo et. al. reported the Engineering Surface State Density of Monolayer CVD grown 2D MoS2 for Enhanced Photodetector Performance. In order to verify dependence of surface states on the responsivity, author observed the surface states were varied by varying temperature and it was observed that upon increment in temperature, the surface states decreases which causes the responsivity values also to decrease. However, a few more clarifications are needed before the manuscript can be considered suitable for publication. A list of other comments that need to be addressed is as follows:

1. In abstract, Author should provide a more concise summary of the key findings and their significance. Avoid introducing new terms or concepts that are not explained in the abstract.
2. Expand the literature review to include more recent research in the field. Consider discussing the latest advancements in photodetector technology and 2D materials, which can highlight the relevance of your study.
3. The manuscript lacks graphical representations of data.
4. The discussion section is relatively brief. Consider expanding on the implications of your findings and how they contribute to the broader understanding of photodetectors.
5. Author should discuss the practical applications and potential advancements enabled by your research.
