# Supplementary material for: Engineering surface state density of monolayer CVD grown 2D MoS2 for enhanced photodetector performance
Source: PLoS One. 2024 Apr 10;19(4):e0297825. doi: 10.1371/journal.pone.0297825 (PMC11006120; doi:10.1371/journal.pone.0297825)
Supplement: Attachment — Submitted filename: Engineering surface state density of monolayer CVD grown 2D MoS2 for enhanced photodetector performance.docx [file pone.0297825.s005.docx]

Engineering surface state density of monolayer CVD grown 2D MoS2 for enhanced photodetector performance.

The manuscript is well written and supported by good experimental and modeling data. Following comments need to be addressed –

1. References in Introduction will be helpful wherein, the author is mentioning the drawbacks of conventional photodetectors.
2. For CVD, what is the temperature?
3. Author mentions about a possibility of defect generation for better performance. Can these defects be quantized? Any surface/vibrational metrology like surfscan or AFM or Raman can be used to quantify the defects.
4. During device performance testing as a function of temperature, what was the maximum temperature observed beyond which the device over heated or the performance saturated. Does this improve the temperature resistance as well?
