# Supplementary material for: Engineering surface state density of monolayer CVD grown 2D MoS2 for enhanced photodetector performance
Source: PLoS One. 2024 Apr 10;19(4):e0297825. doi: 10.1371/journal.pone.0297825 (PMC11006120; doi:10.1371/journal.pone.0297825)
Supplement: Attachment — Submitted filename: Response to Reviewers.docx [file pone.0297825.s006.docx]

**Center for applied physics and advanced technology**

November 21^st^, 2023

RE: Revision Requested for Manuscript reference: PONE-D-23-33109.

Dear Editor,

Thank you for your email dated 31-October-2023 communicating the review on our manuscript reference: PONE-D-23-33109 titled. " Engineering Surface State Density of Monolayer CVD grown 2D MoS_2_ for Enhanced Photodetector Performance". We are glad that reviewers found the manuscript publishable after a revision. We want to thank you and the reviewers for giving our manuscript an opportunity to be considered by *PLOS ONE.* We also appreciate the editors and reviewer’s time and insight comments that have helped us in improving the manuscript. Below please find a detailed delineation of our responses to the comments.

Reviewer(s)' Comments to Author:

Reviewer #1: Bugallo et. al. reported the Engineering Surface State Density of Monolayer CVD grown 2D MoS_2_ for Enhanced Photodetector Performance. In order to verify dependence of surface states on the responsivity, author observed the surface states were varied by varying temperature and it was observed that upon increment in temperature, the surface states decreases which causes the responsivity values also to decrease. However, a few more clarifications are needed before the manuscript can be considered suitable for publication. A list of other comments that need to be addressed is as follows:

Authors reply: Thanks a lot for reviewing the manuscript. The authors are glad that the reviewer found the manuscript publishable after a revision.

1. In abstract, Author should provide a more concise summary of the key findings and their significance. Avoid introducing new terms or concepts that are not explained in the abstract.

Authors reply: Thanks a lot for the suggestion. The authors have elaborately explained the significance of the work in abstract and same is modified and highlighted in updated files.

This study demonstrates the effect of nitrogen doping on the surface state densities (Nss) of monolayer MoS_2_ and its effect on the responsivity and the response time of the photodetector. Our experimental results shows that by doping monolayer MoS_2_ by nitrogen, the surface state (Nss) increases thereby increasing responsivity. The mathematical model included in the paper supports the relation of photocurrent gain and its dependency on trap level. Which states that the increasing the trap density increases the photocurrent gain and the same is observed experimentally. The experimental results at room temperature revealed that nitrogen doped MoS_2_ have a high N_SS_ of 1.63 X 10^13^ states/m^2^/eV compared to undoped MoS_2_ of 4.2 x 10^12^ states/m^2^/eV. The increase in Nss in turn is the cause for rise in trap states which eventually increases the value of photo responsivity from 65.12 A/W (undoped MoS_2_) to 606.3 A/W (nitrogen doped MoS_2_). The response time calculated for undoped MoS_2_ was 0.85 sec and for doped MoS_2_ was 0.35 sec. Finally, to verify the dependence of surface states on the responsivity, the surface states were varied by varying temperature and it was observed that upon increment in temperature, the surface states decreases which causes the responsivity values also to decrease.

2. Expand the literature review to include more recent research in the field. Consider discussing the latest advancements in photodetector technology and 2D materials, which can highlight the relevance of your study.

Authors reply: Thanks a lot for the suggestion. The authors have expanded the state-of-the-art table by including latest advancements in 2D material-based photodetectors and same is included in updated files.

| **Device** | **Detection Range** | **Responsivity**  **(A/W)** |
| --- | --- | --- |
| MoS_2_-BN-G | 400–885 nm | 180 |
| WSe_2_-SnS_2_ | 400–900 nm | 244 |
| ReSe_2_ | 633nm | 95 |
| MoS_2_ | 532nm | 59 |
| In_2_Se_3_ (PVD) | 532nm | 340 |
| GaTe | 254–710nm | 274.3 |
| G/WSe_2_ | 532nm | 350 |
| MoS_2_/SnS_2_ QDs | UV, Visible, NIR | 435 |
| MoS_2_ local Vbg | 532–638nm | 342.6 |
| MoS_2_-ReS_2_ | 555nm | 400 |
| MoS_2_/TiO_2_ nanoflowers | UV | 35.9 |
| Te NWs/ReS_2_ | Visible light (632nm) | 180 |
| BaTiO_3_nanoparticles/ MoS_2_ | UV (365nm) | 120 |
| CuO NWs/MoS_2_ | Visible light | 157.6 |
| **Nitrogen doped MoS_2_ (This work)** | **Visible range** | **606** |

Table: S1 Comparison of responsivities values with existing literature

3. The manuscript lacks graphical representations of data.

Authors reply: Thanks a lot for the comment. Authors have tried incorporating changes in the graphs to have a better representation of the data. The same has been updated in the revised manuscript.

4. The discussion section is relatively brief. Consider expanding on the implications of your findings and how they contribute to the broader understanding of photodetectors.

Authors reply: Thanks for the comment. The authors have added a paragraph regarding the implication of the finding for the general understanding of the photodetector which explains the significance of doping, how it introduces traps and vary the surface states and its effect in the photodetector performance. The same is explained clearly and highlighted in modified file.

5. Author should discuss the practical applications and potential advancements enabled by your research.

Authors reply: Thanks for the query. One of the potential advancements is the observation that the surface states increase the responsivity of the photodetector, and that the responsivity of the photodetector can be tuned by surface states. The practical applications that could be achieved would be visible light communication, healthcare (SpO_2_ monitoring), and security applications (human motion monitoring). The same has been added in the revised manuscript.

Reviewer #2:

1.The author showed the effect of N doping in MoS_2_. The mathematical models seem adequate but paper lacks explanation of the measurement techniques in detail. It would have been good to compare experiment result with mathematical model.

Authors reply: Thanks for the comment. The mathematical model given in the paper explains the relation of photocurrent gain and its dependency on trap level. It is worthy to note that the motivation of the mathematical model was not to compare the experimental results but rather to have a more concrete explanation to the observations from the experimental data. The mathematical model suggests that the increasing the trap density increases the photocurrent gain which also is observed experimentally. So, the mathematical model acts as a support to claim the results from the experimental observations. We humbly request the reviewer to exempt the comparison of experimental result with the mathematical model as that itself would be a different work and the mathematical model needs to be modified for different parameters for its convergence.

Reviewer #3:

1.What is the role of surface states in enhancing the responsivity of photodetector devices, especially when doped with nitrogen?

Authors reply: Thanks for the query. Upon doping the monolayer MoS_2_ with nitrogen, the surface states are introduced as doping predominantly is a surface phenomenon. The introduction of surface states introduces extra energy levels near the conduction band which increases the carrier lifetime thereby increasing the photocurrent gain and the responsivity. The same is also explained by the mathematical model in the paper. As per the reviewer suggestion, the authors have added a line about the same in the revised manuscript.

2. How does varying the density of surface states through nitrogen doping affect the performance of a monolayer MoS_2_ photodetector?

Authors reply: Thanks for the query. As mentioned, the density of surface states increases the carrier lifetime of the photogenerated carriers and as the surface state density increases, the lifetime of carrier increases before recombining thereby increasing the responsivity.

3. What are the practical applications for photodetectors with significantly enhanced responsivity, such as the nitrogen-doped MoS_2_ device described in the research?

Authors reply: Thanks for the query. One practical application with high responsivity would be a reliable visible light communication and also for healthcare application such as SpO_2_ detection.

4. What are the implications of the temperature-dependent nature of surface states on the responsivity of photodetector devices, and how can this be optimized for specific applications?

Authors reply: Thanks for the query. The authors have utilized temperature as one of the parameters to vary/tune the surface states to prove the dependance of the surface states on the responsivity of the photodetector. As rightly pointed out by the reviewer, the temperature dependent nature of the surface states will definitely give rise to the dynamic responsivity which need to be taken into account for specific applications. Further, authors would also like to state that the surface states would actually be modified via doping concentration in real scenario and not by varying the temperature. The temperature variation of the responsivity will be taken into account for the specific application.

5. How does the presence of traps, created by variations in surface states, impact the recombination of carriers in the photodetector and its overall performance?

Authors reply: Thanks for the comment. As the surface states are varied (increases), there would be more energy levels created at the bottom of the conduction band which would avoid recombination and also increase the carrier lifetime and thereby increasing the responsivity. Since there are states below the conduction band due to the introduction of the surface states so the photogenerated carries will reside in those energy levels before it recombines which eventually reduced the recombination probability of the photogenerated carriers.

6. Can the findings and techniques described in this research be extended to improve the responsivity of photodetector devices using other materials or in different wavelength ranges?

Authors reply: Thanks for the query. Yes, we believe that the technique can be used as a universal approach of improving the responsivity of the photodetector. However, it should be noted that one should check the compatibility of different dopants with material of choice as it wouldn’t be possible to have same dopant with other material.

7. What challenges and limitations might researchers encounter when attempting to implement the methods described in this study for practical device applications?

Authors reply: Thanks for the interesting query. The challenges and limitations would be:

1. Controlled doping for controlled responsivity

2. As responsivity increases the response time tend to decrease. Hence to have a perfect balance of responsivity and response time is essential for practical applications

3. The growth of monolayer MoS2 needs to be optimized for having controlled n-type behavior (due to sulfur vacancies). The sulfur vacancies will decide the density of the surface states.

4. Finally the passivation of the device to have least interference with the environmental conditions which might affect the responsivity.

The same challenges have been incorporated in the manuscript.

Reviewer #4: The manuscript is well written and supported by good experimental and modelling data. Following comments need to be addressed.

Authors reply: Thanks a lot for reviewing the manuscript. The authors are glad that the reviewer found the manuscript publishable after revision.

1. References in Introduction will be helpful wherein, the author is mentioning the drawbacks of

conventional photodetectors.

Authors reply: Thanks for the comment, The authors have added the corresponding references.

[1] H. Ren, J. De Chen, Y.Q. Li, J.X. Tang, Recent Progress in Organic Photodetectors and their Applications, Adv. Sci. 8 (2021) 1–23. <https://doi.org/10.1002/advs.202002418>.

[2] S. Mathew, K.K.S. Chandu, S. Halder, G. Polumati, C. Chakraborty, P. Sahatiya, S. Pal, Band alignment and charge transport study of Au nanoparticles decorated over MoS2/MoSe2 hybrid heterostructure for plasmon enhanced photodetection, Mater. Sci. Semicond. Process. 156 (2023) 107302. <https://doi.org/10.1016/j.mssp.2022.107302>.

[3] Polumati, G., Martínez, B. A. M., Kolli, C. S. R., Selamneni, V., Salazar, M. F., Sanchez, D. E., ... & Sahatiya, P. (2023). Band type engineering using different stacking configurations of anisotropic and isotropic monolayer transition metal dichalcogenides. *2D Materials*, *10*(4), 045032. https://doi.org/10.1016/j.mssp.2022.107161.

[4] S. Cai, X. Xu, W. Yang, J. Chen, X. Fang, Materials and Designs for Wearable Photodetectors, Adv. Mater. 31 (2019) 1–15. https://doi.org/10.1002/adma.201808138.

2. For CVD, what is the temperature?

Authors reply: Thanks for the comment, the authors have performed CVD at 800 °C.

3. Author mentions about a possibility of defect generation for better performance. Can these

defects be quantized? Any surface/vibrational metrology like surfscan or AFM or Raman can be

used to quantify the defects.

Authors reply: We thank the reviewer for the question. The quantification of defects in layered semiconductors requires highly specialized facilities like HR-STEM or UH-STM. Unfortunately, with our current characterization tools, it is not feasible to provide an estimate of the number of defects in the MoS_2_ flakes. However, we found differences between the undoped and doped Raman and PL spectra of the MoS_2_ to show the impact of doping.

First, it can be noticed that the N_2_-doped spectrum exhibits a blue shift in the E2g mode compared to the undoped MoS_2_ sample, which can be ascribed to the formation of Mo-N bonding. The discrepancy becomes more pronounced in photoluminescence, where the PL emission of the N_2_-doped sample significantly intensifies compared to the as-grown MoS_2_ flakes, due to the incorporation of nitrogen atoms into sulfur vacancies.

4. During device performance testing as a function of temperature, what was the maximum temperature observed beyond which the device over heated or the performance saturated. Does this improve the temperature resistance as well?

Authors reply: Thanks for the interesting comment. The authors have noticed that the maximum temperature that the device can reach was 65°C beyond which it was observed that the device current and device resistance saturates.

-----x---

Sincerely,

Sincerely,

Dr. Andrés De Luna Bugallo.

Senior Researcher

Centro de Física Aplicada y Tecnología Avanzada,

Universidad Nacional Autónoma de México (UNAM),

A.P. 1-1010, Querétaro, Qro., C.P. 76000, México

e-mail: [**aluna@fata.unam.mx**](mailto:aluna@fata.unam.mx)
